# An Introduction to Nonlinear Integrated Photonics: Structures and Devices

**DOI:** 10.3390/mi14030614

**Published:** 2023-03-07

**Authors:** Luigi Sirleto, Giancarlo C. Righini

**Affiliations:** 1 National Research Council (CNR), Institute of Applied Sciences and Intelligent Systems (ISASI), Via Pietro Castellino 111, 80131 Napoli, Italy; 2 National Research Council (CNR), Institute of Applied Physics “Nello Carrara” (IFAC), Via Madonna del Piano 10, Sesto Fiorentino, 50019 Florence, Italy

**Keywords:** photonics devices, nonlinear photonics, integrated photonics, photonic structures, optical materials, all-optical signal processing, all-optical computing, all-optical communications, all-optical digital devices, signal amplification and frequency conversion, all-optical signal regeneration, supercontinuum generation, microcomb generation

## Abstract

The combination of integrated optics technologies with nonlinear photonics, which has led to growth of nonlinear integrated photonics, has also opened the way to groundbreaking new devices and applications. In a companion paper also submitted for publication in this journal, we introduce the main physical processes involved in nonlinear photonics applications and discuss the fundaments of this research area. The applications, on the other hand, have been made possible by availability of suitable materials with high nonlinear coefficients and/or by design of guided-wave structures that can enhance a material’s nonlinear properties. A summary of the traditional and innovative nonlinear materials is presented there. Here, we discuss the fabrication processes and integration platforms, referring to semiconductors, glasses, lithium niobate, and two-dimensional materials. Various waveguide structures are presented. In addition, we report several examples of nonlinear photonic integrated devices to be employed in optical communications, all-optical signal processing and computing, or in quantum optics. We aimed at offering a broad overview, even if, certainly, not exhaustive. However, we hope that the overall work will provide guidance for newcomers to this field and some hints to interested researchers for more detailed investigation of the present and future development of this hot and rapidly growing field.

## 1. Introduction

In recent decades, the overall trend of research and development (R&D) towards miniaturization of devices and systems begun with microelectronics has led to continuous growth in integrated photonics. Integrated optic and photonic chips have huge potential for low-cost, scalable production, and high-density components integration. A major challenge of integrated photonics has been constituted by the lack of a unique base material, playing a similar role as silicon in microelectronics. In contrast to electronic integrated circuits (ICs), photonic integrated circuits (PICs) are currently fabricated on different constituent materials. In order to integrate multiple photonic functions, different building blocks are fabricated using monolithic or hybrid integration technology over a single substrate and connected via waveguides [1,2,3,4]. Photonic building blocks can be passive (e.g., couplers, switches, modulators, and multiplexers) or active (e.g., amplifiers, detectors, and lasers); nonlinear functionalities may be easily added, exploiting the nonlinear properties of the materials and suitable light-confining structures. As efficiency of nonlinear optical interactions drastically scales up with the power density of an optical wave, it had to be expected that combining integrated photonics and nonlinear photonics would produce groundbreaking results [5,6,7,8,9,10,11,12]. In this review, we focus on three main areas of application: all-optical computing, all-optical processing, and nonlinear photonics sources. 

In all-optical computing, the key strength provided by optical technologies is parallelism of information transfer and processing onto multiple wavelength channels. Parallel access to information points is permitted due to the capability to use light waves of distinct wavelengths within the same device. In addition, photon being the information carrier, there is no propagation delay in different parts of the optical system. 

Implementation of logic operations using a photon signal is a very challenging frontier of research because of the fundamental requirement of very efficient light-control. Ultrafast all-optical switch is a fundamental component for all-optical computing. It can be defined as a structure with a pump light-controlling the ON/OFF transition of the signal light. Optical devices, performing digital functions, are expected to fulfill the following criteria: (1) ultra-compactness; (2) low power consumption (∼fJ/bit); (3) high-speed operation (scalable beyond 100 Gb/s); (4) parallel operation on multiple wavelength channels, reducing the need for large fan-outs and redundant parallel processing structures; (5) preservation of information (e.g., phase) carried in the optical domain, usually lost in optical–electronic conversion; (6) the ability to transparently process a data channel regardless of its data rate or its modulation format; (7) scalability; and (8) cascading [13,14,15,16,17,18]. Unfortunately, as underlined by Grinblat et al. in 2020, at that date, no known structure behaved as an ideal switch [18]. 

The basic building block of an optical flip-flop device is the optical bistable switch. In this device, the output of the system takes on one of two possible states depending on the states of the inputs. Optical bistable operation permits optical read–write memory operation, opening the possibility of an integrated optical logic circuit on a single chip. A typical way of forming a bistable optical device is to place a saturable absorber inside a resonator. As the input intensity is increased, the field inside the cavity also increases, lowering the absorption of material and thus increasing the field intensity further. If the intensity of the incident field is subsequently lowered, the field inside the cavity tends to remain large because the absorption of the material system has again been reduced. However, even if single bistable switches have already been demonstrated on different platforms, it is worth noting that the next big challenge is realization of a complex system where several bistable switches are connected in tandem and in parallel [19,20,21,22,23,24,25,26,27]. 

Nowadays, almost all data flow, including internet, phone calls, etc., goes through fiber optic transmission lines, and the field of communications continues to expand to higher data rates and shorter delays to allow more capacity. The demands of the modern world are for high-speed optical communication and interconnection. The increase in the amount of data available presents both opportunities and problems, leading to “big data” (i.e., large, diverse sets of information that grow at ever-increasing rates) issues. Storing and handling big data may be difficult using traditional techniques; such a new challenge may be faced by using photonic devices for massive parallel processing and nonlinear photonic devices for ultrafast handling [28,29,30]. Today, in optical networks, data are encoded on photons for transmission, while information processing is often carried out through optical–electrical–optical (OEO) conversions. In the last 20 years, with the aim to assist/replace some of the electronic modules used in network routers, optical signal processing systems (OSPs) have been investigated. OSP refers to a broad range of techniques with the aim to process and manipulate the signal, i.e., amplitude, phase, wavelength, and polarization of optical waves, directly in the optical domain. Optical signal processing techniques employ a wide range of devices and various nonlinearities to achieve multiple network functionalities. Widely used functionalities demonstrated in nonlinear photonic circuits (PICs) include wavelength [31,32,33,34,35] and format conversions [36,37], routing [38,39], phase-sensitive amplification [40], optical multiplexing and demultiplexing [41,42,43,44,45], optical memory [46,47], all-optical tunable delay [48,49], and signal regeneration [50,51]. Thus far, most of the existing OSP research has relied on third-order nonlinearities, such as four-wave mixing (FWM), self-phase modulation (SPM), and cross-phase modulation (XPM) [52,53,54,55,56,57,58,59]. 

Current optical networks are mostly based on time-division-multiplexing (TDM) and wavelength-division-multiplexing (WDM). In the former, multiple relatively low-bit-rate streams of data with the same carrier frequency are interleaved to create a single high-bit-rate stream, while the latter involves simultaneous propagation of multiple data signals, each at a different wavelength in a single optical line. Wavelength-division-multiplexing (WDM) techniques offer very effective utilization of the fiber bandwidth directly in the wavelength domain. A first issue of current optical networks is wavelength-blocking. In order to overcome it, a fundamental piece is represented by wavelength conversion devices. They can be obtained using different nonlinear effects, such as FWM or XPM [31,32,33,34,35]. Another issue is to increase transmission bandwidth, and an option is to combine TDM and WDM. In this process, demultiplexing an ultra-high-data-rate time-multiplexed signal to speeds receivable through electronics is achieved by wavelength conversion. Specifically, information multiplexed in the time domain through optical time demultiplexing (OTDM) can be converted into parallel lower-data-rate wavelength or spatial channels. This process has been achieved utilizing FWM, whereby a relatively low repetition rate pump switches out temporally multiplexed channels by converting them to new wavelengths (the idler in the FWM process) [41,42,43]. There are, of course, other routes that can be followed to increase transmission bandwidth and/or to develop advanced processing chips. The goal of increasing bandwidth density of on-chip interconnects without increasing the number of waveguides, waveguide crossings, and chip footprint, for instance, may be reached by exploiting mode-division-multiplexing (MDM) in conjunction with WDM [44]. Polarization-division-multiplexing (PDM) and orthogonal frequency-division-multiplexing (OFDM) are two other effective methods to increase the spectral efficiency of a communication system [45].

When signal modulation rate increases, signal degradation in the optical channel caused by dispersion, nonlinearity, and noise becomes a critical issue. Conventionally, signal regeneration in an optical system is performed through optical–electrical–optical (OEO) conversion, in which a weak and distorted signal is detected, restored in electronics, and retransmitted onto an optical fiber. Regenerators are designed to increase system performance, reduce data degradation, and enhance signal-to-noise ratio (SNR) for higher link capacity. In general, regenerators perform three signal processing functions: (1) reamplifying, (2) reshaping, and (3) retiming the signal. When the data rate is becoming higher and higher (towards 100 Gb/s), optoelectronic regeneration schemes will be very hard to implement or even impossible. Thus, all-optical regeneration, either 2R (re-amplification, re-shaping) or 3R (re-amplification, re-shaping, re-timing), has become a key technology to improve signal quality [50,51]. 

Nonlinear optical sources provide an outstanding example of new possibilities offered by integrated nonlinear photonic chips, such as generation of new classes, named supercontinuum generation [60], and microcombs [61], which are capable of generating coherent, ultra-broadband light sources that cannot be produced in linear photonic systems. Supercontinuum generation is a device functionality that has important applications in many areas of photonic integrated circuits, particularly in WDM applications. As an example, it could be beneficial to use a single broadband laser source, select by filtering specific wavelength channels, and then modulate these channels instead of using a separate laser for each wavelength. 

Optical frequency combs (OFCs) are often referred to as optical rulers: their spectra consist of a precise sequence of discrete and equally spaced spectral lines that represent precise marks in frequency. This discrete ensemble of equally spaced laser frequencies that distinguish OFCs from other light sources is the spectral counterpart of the regular train of short pulses emitted by mode-locked lasers. The OFCs solve the challenge of directly measuring optical frequencies and are now exploited as the most accurate time references available, ready to replace the current standard for time. Laser frequency combs can provide integrated sources of mutually coherent laser lines for terabit-per-second transceivers, parallel coherent light detection, or photonics-assisted signal processing. 

Readers interested to delve into the main physical processes involved in nonlinear photonics applications and the most appealing materials are referred to a companion paper [62]. The focus here is to present some examples of nonlinear integrated photonic devices, with a brief overview of the types of optical integrated structures and the most common material fabrication platforms. In the next section, planar and three-dimensional geometry structures are introduced; in the third section, material platforms are discussed, and, in the last section, advances in nonlinear integrated photonic devices that provide an idea of the high potential for practical applications and future challenges are reported and discussed.

## 2. Integrated Photonic Structures 

Influenced by the general trend of sciences towards the nano-world, development of integrated photonics, begun with cm-long circuits, went through microphotonics and finally came down to nanophotonics. Guided wave structures, resulting from the coupling between an electromagnetic field and some resonance, are the basic blocks that have accompanied this process. 

### 2.1. Optical 2D and 3D Waveguides

**Figure 1:**
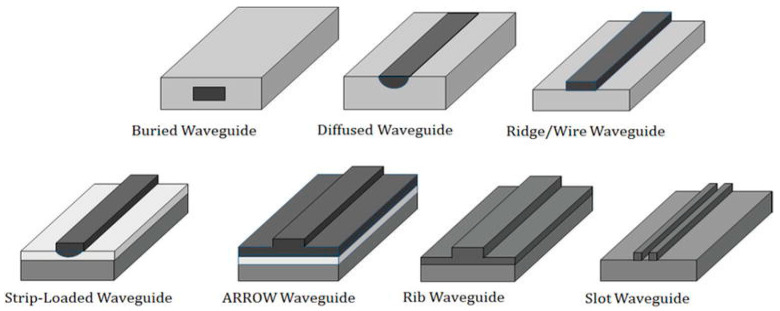
Schematic representation of various channel waveguides. Adapted from [63] under Creative Commons License 3.0.

Resonance can be geometric, as occurs in integrated optical waveguides where constructive interferences result in guiding light due to total internal reflection. Thus, optical waveguides can be classified depending on geometry. Planar (2D) dielectric waveguides are built of layers of high- and low-refractive-index materials, providing confinement only in vertical direction. Nonplanar (3D) waveguides, providing confinement in two directions, can have different cross-sections, such as ridge, rib, stripe-loaded, or buried; slot structures, moreover, can have different forms of guiding core surrounded by cladding material (see Figure 1 for some examples) [63]. Correspondingly, the most suitable fabrication methods must be selected (e.g., high- or low-energy diffusion, thin-film deposition, direct laser writing). The need of efficient coupling to other integrated components makes the channel waveguide the most commonly used structure. Numerical and analytical methods are widely available to optimize the design of waveguides and circuits [64].

The next step, from cm to micrometer scale, was motivated by the wish to investigate light behavior on the microscale and its interaction with micro-objects. The key challenges were a reduction in size of optical devices and improvement in their performances. The main goal was to go beyond the limit of integrated optics, offering a reliable platform for dense integration [65]. 

### 2.2. Microresonators and Photonic Crystals

During recent decades, the fast growth of micro-scale fabrication techniques has enabled successful demonstration of various types of microphotonic devices; special attention has been addressed to microcavities [66,67], such as whispering gallery modes resonators (WGMR) and photonic crystals (PhC) [68,69], which combine small modal volume with very high optical quality factor (Q). In these microphotonic devices, due to their reduced size, photons are trapped in small volumes close to the diffraction limit for sufficiently long times so that these photons strongly interact with the host material, creating enhanced nonlinear effect and significant reduction in their power threshold [66,67,68,69]. 

**Figure 2:**
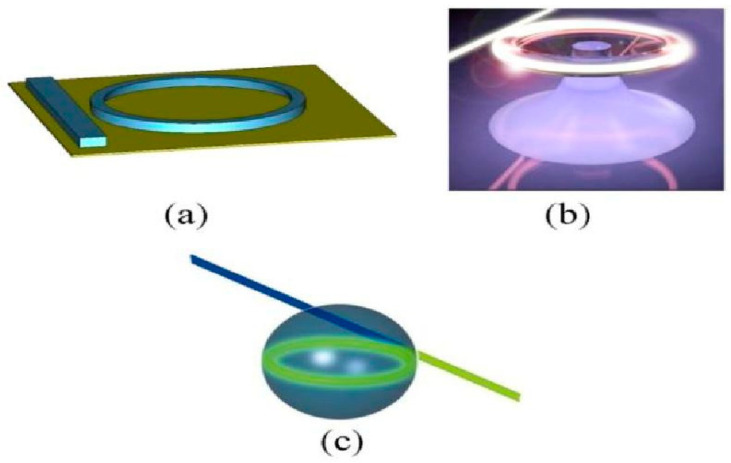
Different types of optical microcavities: (**a**) micro-ring resonator, (**b**) micro-toroid resonator, (**c**) micro-sphere resonator. Adapted from [76] under Creative Commons License 3.0.

WGMRs have shown high mode stability and ultra-high-quality factors Q, up to 10^11^. These resonators are excellent platforms for fundamental and applied studies of nonlinear processes due to their long photon lifetimes (temporal confinement) and their small mode volumes (spatial confinement) [70,71,72,73,74]. Temporal and spatial confinement have made possible optical frequency conversion with low-power continuous-wave (CW) lasers with powers ranging from micro-watts to milliwatts. However, the high circulating intensities inside a WGMR are not a sufficient condition for efficient harmonic generation, parametric, and hyper-parametric oscillations: these phenomena require fulfilling phase and mode matching and energy conservation conditions [71,75]. Figure 2 shows different types of optical microcavities. It must be underlined that, even if more difficult to integrate in a compact structure, microspherical resonators play an important role in nonlinear photonics [70,71,76].

Photonic crystals provide an excellent building block for photonic integrated circuits and for enhancing a variety of nonlinear optical processes as well [77,78,79,80,81]. A strong advantage of PhC cavities with ultra-small-mode volume V_m_ is that quality factors can be relaxed while still achieving excellent Q/V_m_ values; despite the fact that the quality factor is inversely proportional to the linewidth of the cavity, both high-bandwidth and low-threshold all-optical processes can be realized in a single system. Integration issues, of course, must be considered in the case of PhCs as well, especially for 3D structures [80].

**Figure 3:**
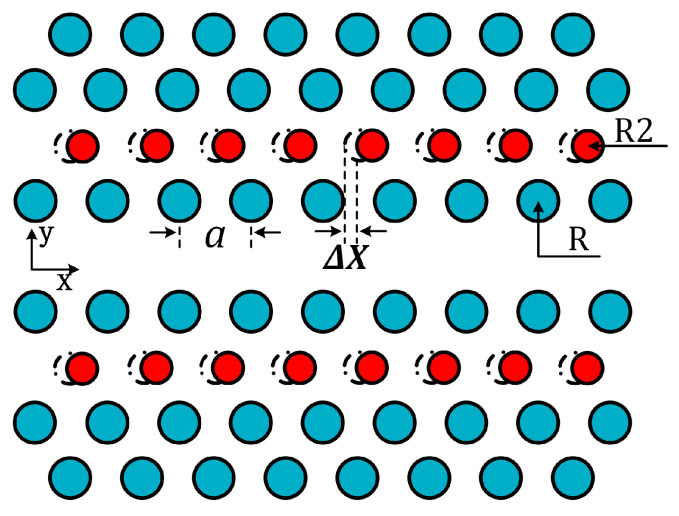
Structure of a photonic crystal waveguide, with air holes represented by blue and red circles. Simulations were made by considering standard silicon-on-insulator air-bridge PhCW of 220 nm thickness, lattice period 400 nm, and radius R of the first air-holes row 100 nm. By adjusting the radius R2 and lattice positions ΔX of the second air-holes rows, a very wide flat band larger than 50 nm could be obtained. Reproduced from [81] under Creative Commons License 3.0.

Figure 3 shows the design of a dispersion-engineered slow-light photonic crystal waveguide (PhCW); the blue circles represent the air-holes in a standard single-line defect (W1) PhCW with a lattice constant a, while the red circles represent the engineered air-holes, with radius decreasing from R to R2 and horizontally shifted from the dashed circles by ΔX [81].

Guided waves can also result from coupling between an electromagnetic field and resonance related to material properties. For example, plasma resonance associated with the electron gas in a metal can be coupled to an electromagnetic field via the interaction between the field and the charges, leading to surface plasmon. These surface waves, propagating along the dielectric–conductor interface, are evanescently confined in the perpendicular direction due to very shallow penetration of the electromagnetic field into the metal. Plasmonic waveguiding allows breaking the diffraction limit of light, opening possibilities for subwavelength light confinement. To increase integration density and compactness of photonic structures, many different geometries of plasmonic waveguide have been investigated in recent years, including interferometers and ring resonators [82] and periodic metal structures [83]. However, in all cases, energy dissipation in closely spaced metal layers causes high losses, limiting the propagation length to a few micrometers.

Nanophotonics is another fascinating field, investigating the light behavior on the nanometer scale and its interaction with nanometer-scale objects [84,85,86]. Major demand in the near future is expected for devices that should allow to control light with light in a very thin nanoscale layer or in a single nanoparticle of nonlinear material. During the past few decades, a significant number of nanomaterials have shown notable optical properties, motivating fabrication and design of nanoscale photonic devices [87,88]. 

Electromagnetic metamaterials are artificial media in which subwavelength electromagnetic constituents replace atoms as the basic structural elements to control light–matter interaction. Many novel phenomena related to metamaterials are due to optical magnetism, observed in specifically designed artificial subwavelength structures, even when such structures are made of non-magnetic materials. The most popular constitutive elements of metamaterials are made of metals, where free electrons oscillate back and forth, creating effective loops of current, thus inducing an efficient magnetic response. Plasmonic nanostructures support field localization due to localized surface-plasmon resonances with multipolar electric-type characteristics. The plasmonic resonances, generally, result in high field enhancement but at the price of higher absorption losses due to free-carrier absorption and reduced optical damage thresholds [89,90]. 

In order to control light–matter interactions at the nanoscale, the most disruptive strategy would be to replace metals by all-dielectric nanoparticles with high refractive index [91,92,93], providing fine control over the light features (amplitude, phase, and polarization). Dielectric nanoparticles support an alternative mechanism of light localization in subwavelength optical structures via low-order dipole and multipole Mie resonances [94]. They may generate a magnetic response via displacement current contribution, playing a crucial role in the realization of the unique functionalities of meta-atoms, also driving novel effects in the fields of metamaterials and nanophotonics. 

It can be mentioned that important steps towards ultra-compactness of photonic circuits have been made very recently via either anisotropic metamaterials [95] or photonic inverse design [96,97,98]. As an example, a polarization beam splitter (PBS), which is an important device in PICs for multiplexing and demultiplexing polarizations, was designed and fabricated on a silicon-on-insulator (SOI) platform using a directional coupler with single-mode waveguides 500 nm wide; the coupling region was designed by tailoring the anisotropic metamaterial, which was composed of identical periodic subwavelength strips. The device, with a 2.5 × 14 µm^2^ footprint, exhibited a low insertion loss of 1 dB, high extinction ratio >20 dB, and wide operational bandwidth >80 nm [98].

**Figure 4:**
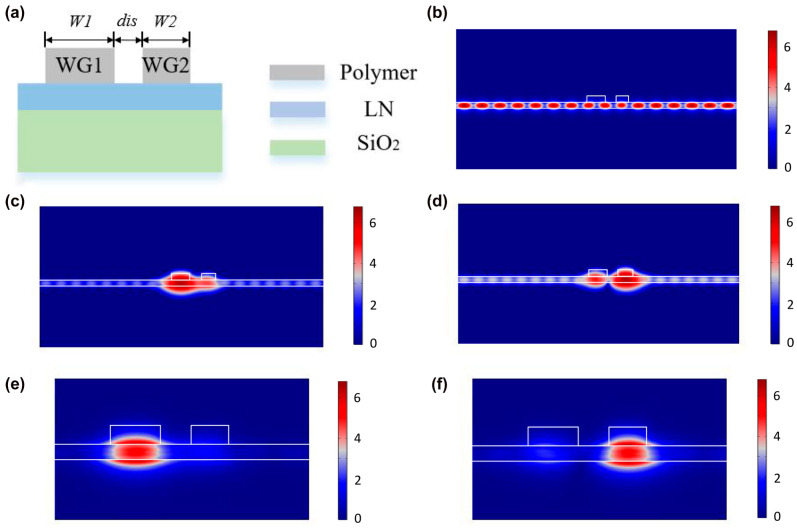
BIC waveguide structure based on polymer strip waveguides (WG1 and WG2) onto a lithium niobate film. Here, WG1 and WG2 are 500 nm thick and LN film is 300 nm thick. (**a**) Cross-section of the structure; (**b**) normalized electric field distribution of the TE continuous mode; (**c**) and (**d**) TM leaky modes. Under proper conditions, the coupling between the TE continuum modes and the TM bound modes can lead to well-confined BIC modes (see (**e**,**f**)). Reproduced from [109] under Creative Commons License.

There is strong interest in exploring interesting physical mechanisms to create the optical resonances in such structures, for example, using guided-mode resonances [99] and bound-states in continuum resonances [100], to enhance local electric fields and consequently amplify the nonlinear optical effects in metamaterials [12,101,102,103,104,105]. Bound states in the continuum (BIC), experimentally observed by Capasso et al. in semiconductor heterostructures grown by molecular-beam epitaxy [106] in 1992, are special wave solutions embedded in a radiative continuum, which, however, remain localized without coupling to the extended waves or radiation. BICs have been observed in photonics by Marinica et al. [107] by using simple structures, such as two arrays of identical dielectric gratings or two arrays of parallel dielectric cylinders [107]. BICs have then been studied in a wide range of material systems, including dielectric photonic crystals, optical fibers, and waveguides; an interesting review was published in 2016 by Hsu et al. [108]. Figure 4 shows an example of BIC structure based on two coupled polymer strip waveguides (WG1 and WG2) onto a lithium niobate (LN) thin film. Etching of LN is not necessary as the strips can be directly patterned through a single e-beam lithography process. When the parallel strip waveguides are placed closely (distance of the order of micrometer), the TE continuum modes exist, as shown in Figure 4b.

Recently, there have been several works on highly efficient second harmonic generation using BIC structures in lithium niobate, which seem to have great potential for development of compact coherent light sources in a broad wavelength range [110,111,112].

**Figure 5:**
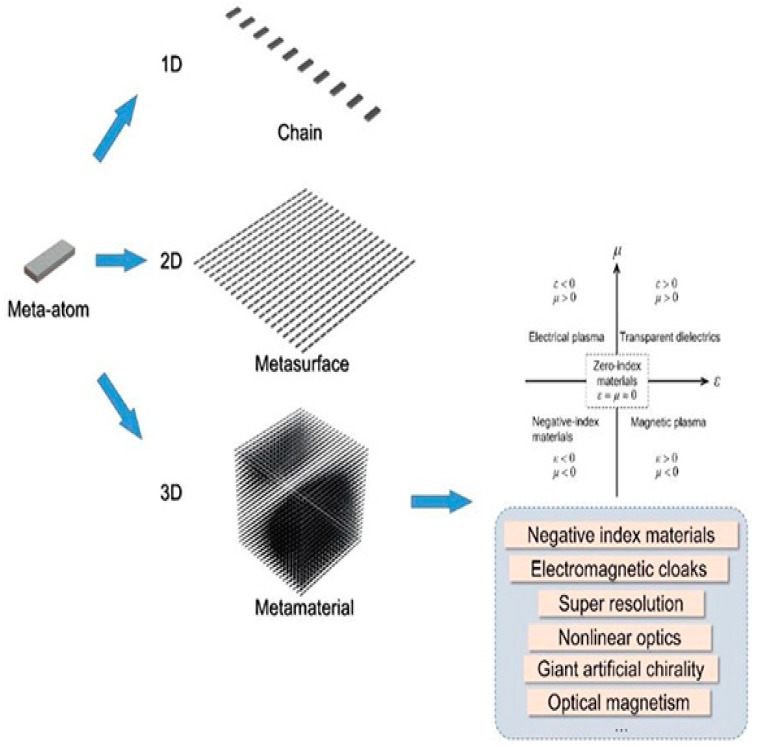
Schematic illustration of meta-atom, 1D chain, 2D metasurface, and 3D metamaterial. Inserts are the representation of the parameter space for permittivity ε and permeability μ and the typical examples of applications of metamaterials. Adapted from [102] under Creative Commons License 3.0.

Figure 5 presents a schematic illustration of some metastructures [102]. 

## 3. Material Platforms for Nonlinear Integrated Photonics

As mentioned in the companion paper [62], numerous materials for integrated nonlinear optics have been investigated. Among them are Si and related materials, such as SiN, a-Si, and SiC; glasses, such as silica, high-index glass, and chalcogenide glasses; III–V semiconductors, in particular AlGaAs and lithium niobate (LN); and recently investigated materials, such as tantalum pentoxide (Ta_2_O_5_) and vanadium dioxide (VO_2_) [62] and references therein. Correspondingly, several material platforms have been developed to achieve the goal of a dense components’ integration. Each platform has its advantages and disadvantages and offers variable nonlinear efficiencies and integration densities depending on the values of the nonlinear coefficients and refractive index contrast. An additional limiting factor is related to losses that are determined by both the quality of the grown material and the maturity of the fabrication process, which affect, for instance, the roughness and absorption of the surface passivation layer. Although in the longer term the growth and fabrication quality for all these materials will eventually level up, there still remains one important limiting factor that clearly separates them regarding high power densities, namely two photon absorption (TPA). TPA is determined by the band gap of the materials and by the working wavelength, which is often in the telecom range due to the historically developed infrastructure of sources, detectors, etc. In this regard, small-band-gap semiconductors, such as Si [113,114,115,116,117] and GaAs [118,119], are fundamentally limited and there seems to be no way around this problem other than to change the working wavelength and all the surrounding infrastructure. 

Silicon photonics research and development has much progressed, and both component performance and integration complexity have made significant steps forward in the past decade [113,114,115,116,117]. Nowadays, silicon-based platforms, in particular silicon-on-insulator (SOI), are among the most mature for PIC realization. The silicon-on-insulator (SOI) platform is a fabrication approach in which a thin silicon layer on top of an insulator layer resides on a silicon substrate. The functional optical elements are situated in the thin top-silicon layer, and the insulator is typically made from SiO_2_. The SOI platform has become the foundation of silicon photonics for several reasons, including strong optical confinement of silicon due to the significant refractive index difference between silicon and SiO_2_, which enables very compact optical devices, and demonstration of much more compact optical waveguides with lower optical propagation loss, better processing yield, low cost, etc.

Among the numerous nonlinear waveguide platforms that have been explored, the group of materials capable of combining both passive waveguides, for light steering and nonlinear manipulation, and active functionalities (laser sources, modulators, and detectors) monolithically on the same chip is III–V semiconductors [118,119]. This has been a strong driving force stimulating development of nonlinear optical devices based on III–V semiconductors and addressing challenges associated with loss mitigation and nonlinearity enhancement in these platforms. The main III–V integrated nonlinear photonic platforms considered to date are GaAs [76] and its AlGaAs [120] derivative, InP and InGaAsP, III-nitrides AlN [121,122], and GaN [123], as well as GaP [124] and its ternary derivative InGaP. We note that AlN [121,122] shows optical properties similar to SiN (refractive index, transparency, and n_2_) but also possesses second-order nonlinearity.

**Figure 6:**
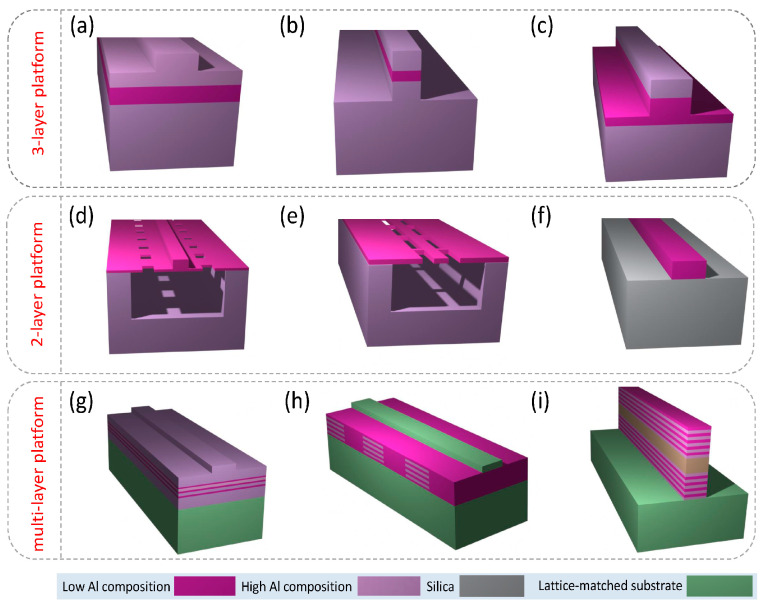
Schematics of AlGaAs platforms and waveguide geometries. (**a**–**c**) 3-layer platform with strip-loaded, nanowire, and half-core waveguides, respectively. (**d**–**f**) 2-layer platforms with suspended nanorib, suspended nanowire, and AlGaAs-OI waveguides, respectively. (**g**–**i**) Multi-layer platform with multi-quantum-well waveguide, modulated-χ^(2)^ waveguide, and Bragg-reflector waveguide, respectively. Reproduced from [120] under Creative Commons License.

Considering AlGaAs, the structures used in NLIP can be divided into three-layer, two-layer, and multi-layer platforms depending on the number of epitaxially grown layers with different material compositions. Figure 6 shows examples of these three AlGaAs platforms; the waveguides sketched in Figure 6h,i are often employed for phase matching of the χ^(2)^ processes. 

In recent years, lithium niobate-on-insulator (LNOI) wafer fabrication process has been rapidly advancing [125,126,127,128,129]. This technology is revolutionizing the lithium niobate industry, enabling higher performance, lower costs, and entirely new devices and applications. Availability of LNOI wafers has sparked significant interest in the platform for integrated optical applications as LNOI offers the attractive material properties of lithium niobate while also offering stronger optical confinement and high optical element integration density that has driven the success of more mature silicon and silicon nitride (SiN) photonics platforms. Many of the key building blocks for highly integrated photonic integrated circuits (PICs) have been established on this platform, including low loss optical waveguides, electro-optical interfaces for ultra-fast modulation, and nonlinear optical elements and resonators. However, further work needs to be completed to make LNOI an attractive and competitive integrated optical platform: (i) optical interfacing to LNOI waveguides has to be improved, reducing fiber-to-chip coupling losses, for example by developing inverted tapers and waveguide tapers on LNOI; (ii) optical gain media need to be demonstrated in this platform, either via bonding already doped lithium niobate in the LNOI wafer fabrication process or by doping the LNOI wafer after fabrication using ion implantation techniques. A further approach could be heterogeneous integration of III–V lasers on LNOI waveguides, which needs to be investigated; (iii) development of photodetectors on LNOI waveguides requires further investigation [125,126,127,128,129].

Current trends in PIC material technologies, however, indicate that there is no waveguide material technology that can address the needs for all the potential applications of PICs. Furthermore, advanced PICs may require the best possible performance of many different photonic elements to achieve the desired functionality, which may not be possible with a single waveguide material technology. However, the fundamental size limit for photonic devices is significantly larger than that of electrical devices. Therefore, integrated photonics has, over the decades, developed at a considerably slower pace than integrated electronics, in integration density as well as total number of devices on a chip. The specific application requirements drive the choice of the most suitable substrate material as each material has its own specific advantageous features and limitations. A solution to overcome this limitation is to integrate different material technologies into a single PIC or package. This approach has the benefit that each material can be used to provide the photonic element functionality for which it is best suited without compromising the functionalities of the other elements in the system. Integration of the different material technologies can occur through two different routes: (i) hybrid integration and (ii) heterogeneous integration. The former is a process that connects two or more PICs or photonic device chips, usually from different material technologies into one single package. This process is, in general, performed at the packaging stage after fabrication of the PIC and photonic device chips. The latter, instead, is a process that combines two or more material technologies into a single PIC chip. This process is generally performed at the early- to mid stages of fabrication of the PIC chip, as in the case of unpatterned III–V thin-films integrated onto pre-processed silicon photonic wafers [130,131,132].

**Figure 7:**
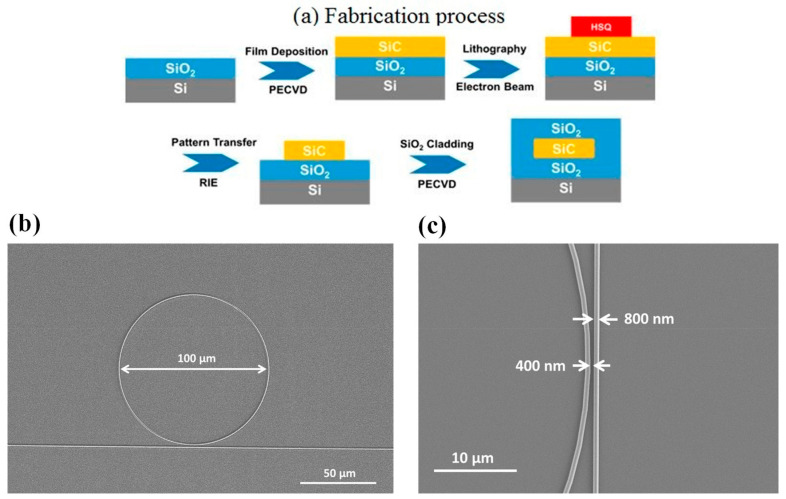
Fabrication of a microring in a 350 nm thick a-SiC film with silica cladding of 3 µm (bottom) and 2 µm (top). (**a**) Schematic process flow; (**b**) SEM micrograph of the microring having 100 µm diameter; (**c**) higher magnification SEM image of the coupling area between the ring and a ridge waveguide. Reprinted with permission from Xing et al., ACS Photonics [134]. Copyright 2019, American Chemical Society.

In order to address the shortcomings of the SOI platform, several novel waveguide platforms have been developing based on heterogeneous integration of other material systems on silicon substrates, with the common requirement of remaining compatible with the complementary metal-oxide-semiconductor (CMOS) technology [132]. As a more recent example, photonic components in SiC (specifically waveguides, 1D and 2D photonic crystal cavities, microdisk, and microring resonators), based on thin layers of SiC on insulator (SiCOI), have been implemented [133,134]. High nonlinearity and low loss were demonstrated in waveguides and ring resonators fabricated in amorphous SiC (a-SiC) grown directly on silica, using plasma-enhanced chemical vapor deposition, with loss as low as 3 dB/cm, providing a very scalable material growth [133]. The intrinsic quality factor of the microring resonator was around 160,000 [133]. Figure 7 shows the fabrication process flow of microring devices (a) and the SEM micrographs of a microring (b) and of the coupling area to a channel waveguide (c).

Much higher quality factors have been achieved by using the 4H polytype of silicon carbide (4H-SiC). For instance, a microring fabricated in high-purity semi-insulating 4H-SiC exhibited a Q = 1.1 × 10^6^, corresponding to a waveguide loss of 0.38 dB/cm [135]. The high field enhancement of this microring (55 µm diameter) enabled demonstration of optical parametric oscillation and optical microcombs. More recently, generation of an octave-spanning microcomb, covering a wavelength range from 1100 nm to 2400 nm, was achieved by using a 36 µm radius microring resonator fabricated in 4H-SiC on a silicon dioxide layer, with intrinsic quality factor above one million [136].

**Figure 8:**
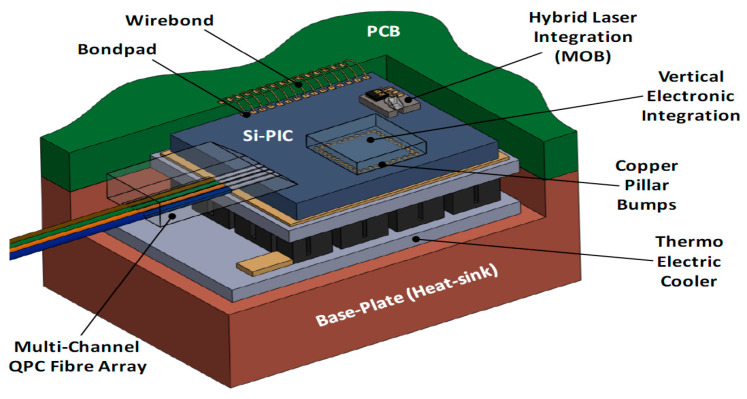
Schematic of a Si-PIC packaged with a multi-channel quasi-planar-coupled (QPC) fiber-array, a hybrid-integrated laser source based on a micro-optic bench (MOB), a vertically integrated electronic integrated circuit (EIC), and a thermo-electric cooler. Electrical connections between the PIC and the printed circuit board (PCB) are made by wire-bonds, while the connections between the PIC and EIC are made using copper pillar bumps (CPBs). Reproduced from [137] under Creative Commons (CC-BY) license.

As a rule, photonic packaging of PICs is much more challenging and much more expensive (at least to date) than electronic packaging since it requires robust high-precision alignment of optical components and stringent real-time temperature control. This is one of the reasons why huge development of commercial integrated photonic devices is still lacking. Figure 8 may provide an idea of the technical complexity of the optical, electrical, thermal, and mechanical interconnections inside and outside a PIC [137].

## 4. Nonlinear Photonics Devices

Development of material platforms described in the previous section has enabled design and implementation of several effective integrated nonlinear devices. Here, we provide a brief, and certainly not exhaustive, overview of this field; for simplicity, we tried to classify the examples into groups characterized by a common goal, even if quite broad.

### 4.1. All-Optical Digital Devices: Switches, Gates, Flip-Flop Units, and Optical Transistors

Two methods are widely used in all-optical switches. The first one is based on linear interference between two light signals, whose relative optical phase difference determines the logic operation functions. Implementation of this method is simple, but, due to the lack of precise control of the optical phase difference, inherent instability, with low-intensity contrast of output logic states ‘1’ and ‘0’, is experienced. The second method is based on third-order nonlinear optical effects, i.e., on the effect of intensity-dependent refractive index. The advantage of this method is its strong universality, and, in principle, complex all-optical devices could be realized based on it. The main obstacle limiting its application is the intrinsic material bottleneck limitation due to the contradiction between the huge third-order nonlinear coefficient and the ultrafast response time (i.e., the larger the third-order nonlinear coefficient, the slower the response time).

**Figure 9:**
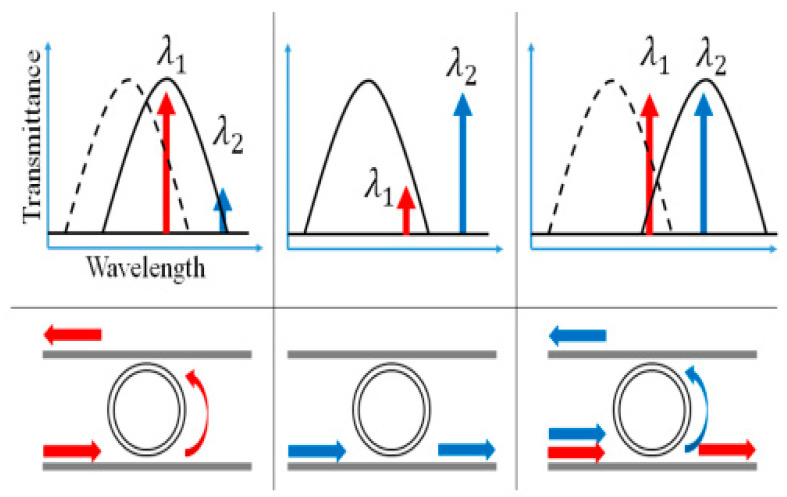
Schematic illustration of an all-optical switch made of an add–drop microring resonator. The dotted line represents the transmittance spectrum of a cold cavity. The solid line is the transmittance when inputs are applied. A resonant shift occurs due to the optical Kerr effect. Two different wavelengths, λ_1_ and λ_2_, are used for the operation. On the left column λ_1_ will drop (high) when only λ_1_ is inputted (high).) On the middle column λ_2_ will not drop (low) when only λ_2_ is inputted (high). On the right column λ_2_ will drop (high) when both λ_1_ and λ_2_ are inputted (high). As a result, λ_2_ can be switched off and on by turning λ_1_ signal on and off. Reprinted with permission from [22] © The Optical Society.

An important enabling technology to implement all-optical switching operation is provided by high Q/V_m_ resonators, Q being the quality factor and V_m_ the mode volume of the cavity. The field intensity enhancement inside the cavity makes it possible to utilize optical nonlinearities at a low input power (a few hundred μW to mW level), achieving picosecond–femtosecond response time and lower energy consumption [13,138]. Microring resonators are among the most widely used configurations for realization of all-optical switch due to their simple design and easy experimental realization [14,16,22,139]. The switching principle, based on nonlinear effects, relies on a shift in resonant wavelength. It is the following: the refractive index variation in the nonlinear material changes the resonant frequency of the microring and/or coupling between the waveguide and the microring. As a consequence, switch control of the signal light output can be enabled. As an example, Figure 9 presents a schematic illustration of an all-optical switch based on a microring resonator.

Photonic crystal cavities are also an important tool in all-optical switching, mostly due to their ability to confine light to extremely small mode volumes [20,140,141,142]. Optical bistable switching by using a high-Q two-dimensional photonic crystal nanocavity was demonstrated experimentally using thermo-optic [20] and carrier [140] effects. In order to make possible an all-optical switch, PC transmission spectrum can change when the pump light is injected into the system and the propagation state can change from allowed to forbidden or from forbidden to allowed. An all-optical switch based on a photonic crystal nanocavity was demonstrated in 2015 [141]. The working principle of the device was based on the resonance wavelength change in the cavity by either the Kerr effect or free carriers. In 2020, Takiguchi et al. [142] demonstrated a picosecond all-optical switch using an InP/InAsP nanowire integrated in a silicon photonic crystal. The line-defect photonic crystal had a Q-factor of 25,000. The reaction time of the switch was 150 ps, while the switching energy was several hundred fJ. 

Photonic crystal chips also lend themselves for large-scale and dense integration of optical memories; this result is obtained by introducing a wavelength-addressable serial integration scheme and exploiting the wavelength-division-multiplexing capability [140]. However, use of a high-Q cavity makes the operation slow because the light charging speed is slow for a cavity having a high Q. Although the carrier effect has enabled fast operation at a few ns [46], a tradeoff between low power and high speed due to the high Q of the cavity remains.

An approach based on nonlinear plasmonic slot waveguides was used to address the well-known tradeoffs between all-optical switching speeds and associated energy requirements [143]. Graphene’s ultrafast saturable absorption (SA), leading to transmission of a signal pulse when a control pulse overlapped with it, was achieved, with an ultrafast response time of 260 fs.

All-optical logic gates and flip-flops are other fundamental elements for optical computing [22], even if still, at the beginning of the last decade, there were doubts about their feasibility and their possibility of competing with microelectronics [144]. Such skepticism was not fully justified, even if, still five years later, realization of an all-optical flip-flop required coupling two optical triode switches made by a nonlinear étalon and gradient-index lenses [19]; to date, however, several all-optical logic devices have been demonstrated [13,25,27,145,146,147,148].

Extremely-low-power all-optical bistability was demonstrated by utilizing silicon PhC nanocavities [46]. Bistability was obtained by the plasma effect of carriers generated by two-photon absorption, with a bistable threshold power of 0.4 mW, a set pulse energy of 74 fJ, and a switching speed of <100 ps. 

Design and fabrication of a large-scale memory in InGaAsP/InP and Si devices were discussed in Ref. [140]. A large number (128) of serially integrated PhC Si-nanocavities were fabricated, and the experimental characterization showed that bistable memory operation was achieved for 105 bits in this chip, with bias power for this operation in the range 79–400 µW. The proposed device involves a parallel configuration, where side-coupled bit memory cavities are placed along a bus waveguide.

Electronic transistors, as the basic unit of logic circuits, have succeeded in supporting large-scale integrated circuits for computers. Any complex logic circuit can express a combination of three basic logic gates, AND, OR, and NOT, and these basic gates can be constructed with a universal transistor. Although electronic logic gates have enabled creation of integrated circuits with high density and functionality, optical logic gates cannot reach the far requirements of large-scale optical computing circuits even today [149]. On the other hand, optical transistors, as the core hardware of optical gates, so far have not been effectively exploited. Moreover, the universal optical transistor does not seem to exist or be practical for optical gates. Many different approaches to optical transistors have been proposed. Much early work was based on optical bistability [150] using nonlinear optical phenomena, mostly in resonators, whereas others worked on the optically controlled switching of light-exploiting single molecules [151] or quantum dots [152].

### 4.2. All-Optical Processing

#### 4.2.1. Signal Amplification

Light amplification over a broad gain bandwidth, in principle, enables generating and processing an array of wavelength channels, leading to significant advancement for densely integrated photonic circuits. In the FWM conversion scheme, typically, a high-power pump laser at $\omega_{p}$ can be used to convert a signal frequency $\omega_{s}$ into a new frequency at $2\omega_{p}-\omega_{s}$. The main advantages of FWM converters are: (1) their sensitivity to both amplitude and phase information; (2) their ability to support ultra-high bit-rates beyond 160 Gbits/s; (3) simultaneous conversions of multiple input wavelengths to multiple output wavelengths; (4) their ability to support advanced modulation formats of input data signals [31,35]. A major limitation is that phase-matching must be maintained over the gain spectrum of interest, requiring careful dispersion engineering. 

As of 2006, a silicon amplifier with net peak on/off gain of 1.9 dB and a broadband gain over a wavelength range of 28 nm through FWM was demonstrated in suitably designed SOI channel waveguides with a length 6.4 mm [153]. In addition, wavelength conversion in the range 1511–1591 nm with peak conversion efficiencies of +5.2 dB was reported as well. A few years later, on-Si-chip mid-infrared gain up to 25.4 dB around a wavelength of 2220 nm with a gain bandwidth exceeding 220 nm was demonstrated [154], providing zero-dispersion at 2260 nm and producing anomalous dispersion of 1000 ps nm^−1^ km^−1^ around the pump wavelength at 2170 nm. In 2017, an optical parametric gain of 42.5 dB, as well as cascaded four-wave mixing with gain down to the third idler, was experimentally demonstrated by FWM in an ultra-silicon-rich nitride (USRN) waveguide [155]. It was attributed to the high photon efficiency achieved through operating above the two-photon absorption edge, representing one of the largest optical parametric gains to date on a CMOS platform.

Another approach to achieve high gain is to exploit hybrid integration. As an example, an efficient semiconductor optical amplifier (SOA) may be introduced into a Si PIC by adding III–V materials as a chip over a selective area of the Si wafer [156]. An unsaturated gain of 12.25 dB/mm with 65 nm of 3 dB bandwidth was measured in an SOA with a 0.95 µm wide waveguide; the InP chip was directly bonded to individual dies on a processed SOI wafer.

#### 4.2.2. Frequency Conversion

In FWM-based converters, the wavelength conversion from the pump to the idler is realized and the information optically encoded onto one wavelength channel can be simply copied onto another wavelength channel. Let us mention a few published results:

(i) C-band wavelength conversion in Si photonic wire waveguides with submicron cross-section was demonstrated by means of nondegenerate FWM (see Figure 10 of [157]). The nonresonant character of the FWM enabled demonstrating frequency tuning of the idler from ∼20 GHz to >100 GHz, thus covering several C-band DWDM channels.
(ii) Conversion bandwidths greater than 150 nm and peak conversion efficiencies of −9.6 dB were also achieved via FWM and appropriate engineering in silicon nanowaveguides [158]. Furthermore, utilizing fourth-order dispersion, wavelength conversion across telecommunication bands from 1477 nm (S-band) to 1672 nm (U-band) was demonstrated with an efficiency of −12 dB.
(iii) A wavelength conversion bandwidth of 190 nm with an efficiency of 21 dB, obtained by FWM in polymer (PMMA)-cladded chalcogenide (As_2_Se_3_) hybrid microwires, was achieved [159]. Wavelength conversion combined with small footprint (10 cm length), low loss (<4 dB), ease of fabrication, and the transparency of As_2_Se_3_ from near-to-mid-infrared regions make the proposed device very promising.

**Figure 10:**
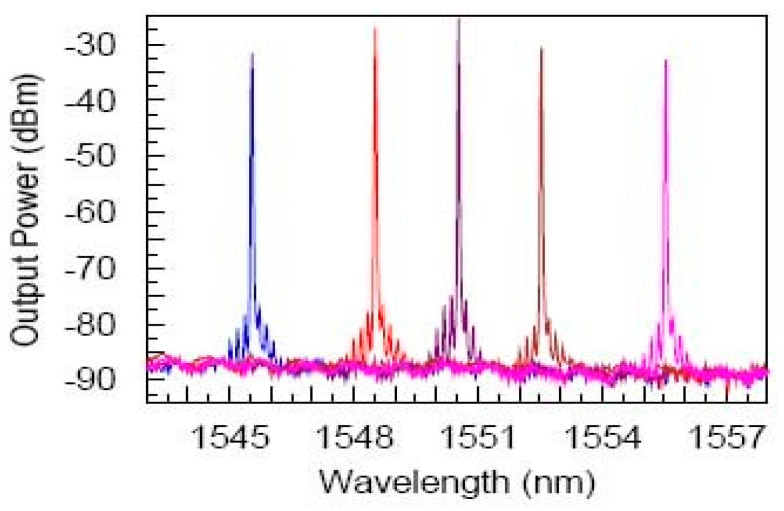
Output spectra from a single-mode silicon photonic wire waveguide with a cross-section of 220 nm × 445 nm and length of L = 4.2 mm fabricated on a SOI. pump (λp~1435 nm), and signal laser sources were multiplexed and launched into the waveguide using a tapered fiber in copropagating configuration; several signal wavelengths (λs = 1545.5, 1548.5, 1550.5, 1552.5, and 1555.5 nm) were used. On each side of the signal wavelength employed, newly generated satellite peaks are clearly seen. Reprinted with permission from [157] © The Optical Society.

The goal of achieving a broad wavelength conversion bandwidth may be achieved acting on group-velocity dispersion (GVD); flattened GVD is also important for effective supercontinuum generation. Silicon waveguides exhibit high nonlinearity, but dispersion flattening represents a difficult task due to the strong light confinement and high waveguide dispersion. One has mainly to work on the design of the cross-sectional dimension of the waveguide, and various solutions have been proposed. Staying with a rib waveguide allows only a few degrees of freedom so that sandwich and slot structures with greater flexibility in geometrical parameters have been designed and tested. Both sandwich and slot waveguides are realized by introducing slots between silicon strips and another material to fill the slot(s); the standard silicon-on-insulator (SOI) technology, however, makes easier fabricating the vertical-slot structure than the horizontal-sandwich one. An early work was based on a hybrid strip/slot silicon waveguide, capable of producing a flattened dispersion of 0 ± 16 ps/(nm·km) over a 553-nm wavelength range (1562 to 2115 nm) [160]. Shortly later, a horizontal double-slot Si waveguide was designed to achieve low and flattened dispersion: simulation indicated that two silica slots at the bottom of the waveguide could produce flattened dispersion from −26 to 21 ps/(nm·km) over a bandwidth of 802 nm, whereas a structure with two silica slots placed at both sides could achieve flattened dispersion from −17 to 23 ps/(nm·km) over a bandwidth of 878 nm (1498 to 2376 nm wavelength) [161]. In 2017, a new structure was proposed, where the field was mainly confined in the silicon core based on silica-filled vertical-dual slots; a spectrally flattened near-zero anomalous group-velocity dispersion covering the whole telecom wavelength range was possible, and, in a wavelength conversion experiment, a 3 dB bandwidth of 76 nm was measured, which was significantly broadened compared to the corresponding strip waveguide [162].

More recently, the problem of data transmission over the C band (1530–1565 nm) closely approaching the nonlinear Shannon capacity limit began to be considered. One of the novel ways to circumvent this limit is to extend WDM beyond the C band, e.g., to the O band (1260–1360 nm). It has been shown that a proper design of an SOI waveguide allows to shift wavelengths from the whole C band into the O band by FWM-based all-optical signal processing, without the need of changing pump laser wavelengths [163]. One year later, the same research group reported the experimental achievement of optical wavelength conversion over the C-to-O-band using multi-modal FWM in highly nonlinear SOI waveguides [164]. The authors claimed that it was an important step towards ultra-high-bandwidth optical communication networks, which utilize the entire low-attenuation infrared wavelength range of a standard single-mode fiber; as a further step towards a complete electronic–photonic-integrated circuit (EPIC), they envisaged the co-integration of the nonlinear signal processor with conventional transmitter- and receiver-units [164].

**Figure 11:**
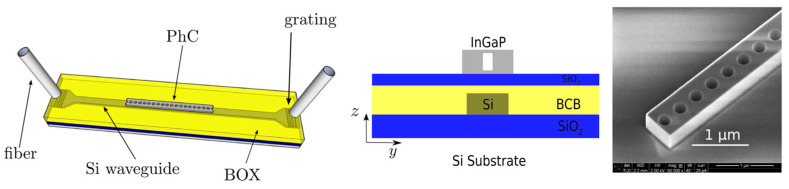
Photonic crystal (PhC) resonator integrated on an SOI platform on Si substrate: 3D sketch (**left**); YZ cross-section with the structure’s layers (**center**); BCB is benzocyclobutene, the adhesive used for bonding; SEM image of the InGaP cavity, 650 nm wide, 290 nm thick (**right**). Reproduced with modifications from [165] under Creative Commons license.

In order to face the difficulty of satisfying the energy and phase matching conditions in a microresonator, an interesting proposal was made by Marty et al. [165], who designed and fabricated a PhC microresonator exhibiting a constant free spectral range. An InGaP cavity, 200 μm long and 650 nm wide, was heterogeneously integrated on an SOI PIC. The structure is shown in Figure 11; the nanobeam cavity is fabricated on top of the Si waveguide using e-beam lithography and hole drilling by inductive coupling plasma (ICP) etching. A stimulated FWM with a −12 dB signal to idler conversion was measured. 

Table 1 presents the characteristics of this PhC device in comparison to ring resonators in different materials (Q_avg_ is the geometrical average of the loaded Q for the different resonance modes; η_NL_ is the nonlinear FWM conversion efficiency). It clearly appears that the PhC on the SOI device, even if with lower conversion efficiency, has the advantage of requiring lower pump power and a remarkably smaller footprint [165].

In order to increase the bandwidth density of on-chip interconnects without increasing the number of waveguides, waveguide crossings, and chip footprint, an option is to exploit mode-division-multiplexing (MDM) in conjunction with WDM [44]. A device based on multiple microring resonators in an SOI wafer, having 0.11 mm^2^ footprint, operated with multiple co-propagating 10 Gb s^−1^ communication signals and reached up to 60 Gb s^−1^ of aggregate bandwidth. It was claimed that, properly designed with five spatial modes and 87 WDM channels, the device could be able to support an aggregate data rate up to 4.35 Tb s^−1^ [44]. 

Using polarization-division-multiplexing PDM, orthogonal-frequency-division-multiplexing OFDM transmission, and a polarization multiple-input multiple-output (MIMO) system at the receiver, a 16 × 52.5 Gb s^−1^ transmission over 4160 km of standard single-mode fiber was demonstrated with 50 GHz WDM channel spacing [45]. 

Advanced modulation formats, such as multiple (M)-order QAM (quadrature amplitude modulation), are fundamental for design of modern high-capacity optical transport networks [171]. In order to enable bandwidth efficient, high-speed transmission on a chip level, integrated optical components that can operate on these modulation formats providing fundamental optical functionalities, such as wavelength routing, wavelength conversion, format conversion, etc., are required. As an example, wavelength conversions of high-order OFDM m-QAM signal based on degenerate four-wave mixing (FWM) process has been demonstrated in a silicon waveguide [172].

Long et al. [173] also used a silicon waveguide: a continuous-wave (CW) pump and a four-channel WDM 16-QAM signal were simultaneously fed into the waveguide through a grating coupler. Owing to the FWM process, the idler takes the information carried by the input WDM 16-QAM signal at the output port. In this way, wavelength conversion of WDM 16-QAM signal using a silicon waveguide was achieved.

In [34], four-wave mixing (FWM)-based wavelength conversion of binary phase shift-keyed (BPSK) and quadrature phase shift-keyed (QPSK) signals at 20-Gb/s bit-rate in a 1-mm long amorphous silicon waveguide was demonstrated with a maximum FWM-efficiency of −26 dB.

The first demonstration of wavelength conversion of higher-order QAM signals at data rates above 100 Gb/s in silicon was reported by Adams et al. [174]. Error-free wavelength conversion of 28 GBaud 16-QAM single polarization (112 Gb/s) signals using FWM in a dispersion-engineered silicon nanowire (SNW) was demonstrated.

In XPM converters, a phase shift is induced on a signal by a pump when it propagates through a nonlinear device. The method is commonly applied along with an interferometric approach, which is able to translate at its output phase variations into amplitude variations. XPM-based wavelength conversion has the advantage of broadband operation, but the limitation is that it can applied only to amplitude modulation formats.

As an example, wavelength conversion in chalcogenide planar waveguides (As_2_S_3_) was achieved in the telecommunications C-band wavelength range via cross-phase modulation, with 5.4 ps optical pulses near 1550 nm [175]. In this device, a pulsed pump source, potentially containing digital data, is directed though the nonlinear medium along with a CW probe beam. The pump beam, inducing a transient chirp on the probe beam via XPM through Kerr nonlinearity, broadens the probe spectra generating sidebands. Using an optical filter, a single sideband can be selected so the output signal at the converted wavelength is modulated in time similarly to the pump pulse. 

Astar et al. presented tunable wavelength conversion of a 10-Gb/s return-to-zero on–off-keyed (RZ-OOK) signal over a range of 20 nm, carried out in a silicon nanowire waveguide using cross-phase modulation (XPM), followed by a tunable filter [176].

**Figure 12:**
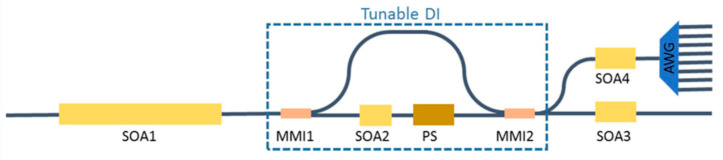
Schematic of an all-optical four-channel processor for NRZ to RZ format conversion to be integrated in an InP monolithic chip. MMI1 splits the signal and MMI2 recombines the signals from the two arms, which have a different phase shift. Operation wavelength is around 1570 nm, which is close to the gain peak of SOAs. Reproduced with permission from [177], 1943-0655 © 2023 IEEE.

**Figure 13:**
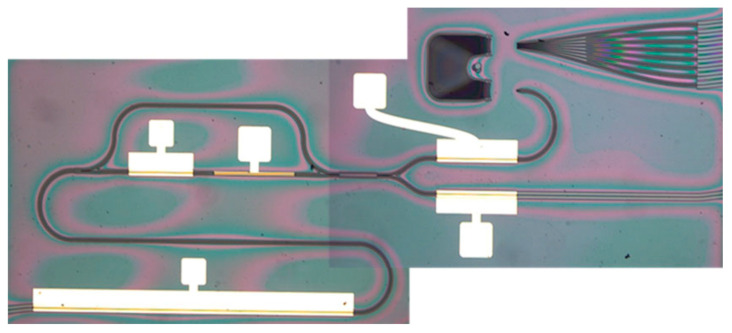
Image by a metallographic microscope of the fabricated InP monolithic chip according to the schematic in Figure 11. Reproduced with permission from [177], 1943-0655 © 2023 IEEE.

All-optical four-channel format conversion from non-return-to-zero on–off-keyed (NRZ-OOK) to return-to-zero on–off-keyed (RZ-OOK) formats, based on semiconductor optical amplifiers (SOAs), was demonstrated in Ref. [177]. The corresponding monolithic InP-integrated chip is sketched in Figure 12: in its 2.0 × 4.6 mm^2^ footprint, it includes a 2.0 mm long SOA1, used for nonlinear XPM and XGM effects, a tunable delay interferometer (DI), two short SOAs, and an arrayed waveguide grating (AWG). The DI, in turn, consists of two multi-mode interference (MMI) couplers and a 400 µm long SOA (SOA3) that compensates the loss introduced by the 500 µm long phase shifter (PS). An image of the chip obtained by a metallographic microscope is shown in Figure 13. The device was operating the conversion process at 40 Gb/s and with an average power penalty at BER (bit error rate) of 1 × 10^−9^ of the four channels less than 0.5 dB. The authors claimed that this chip has the potential to be used for other parallel all-optical signal processing, such as multichannel wavelength conversion, parallel signal regeneration, and so on [177].

#### 4.2.3. All-Optical Signal Regeneration

**Figure 14:**
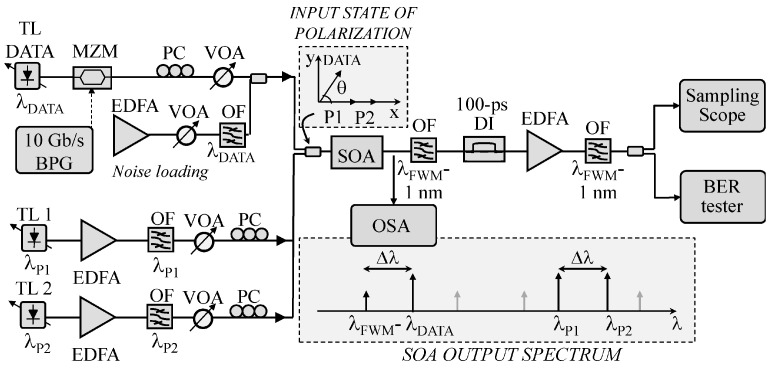
Sketch of the experimental setup for characterization of a SOA-based polarization-independent all-optical regenerator for DPSK data. The principles of operations are schematized within the two dashed boxes. (TL: tunable lasers; MZM: Mach–Zehnder modulator; BPG: bit pattern generator; EDFA: Erbium-doped fiber amplifier; PC: polarization controller; VOA: variable optical attenuator; OF: optical filter; DI: delay line.) Reproduced from [181] under Creative Commons license.

Most of the work completed so far on all-optical signal regeneration has exploited the nonlinear properties of optical fibers [178,179] or semiconductor optical amplifiers (OAS) [180,181,182]. Figure 14 shows the experimental setup for characterization of a polarization-independent differential phase shift keying (DPSK) regenerative wavelength converter [181]. A 10 Gb/s DPSK data stream at λ_DATA_ is generated by modulating the output of a tunable laser (TL) with a Mach–Zehnder modulator (MZM) driven by a bit pattern generator (BPG). The modulator output is then coupled with an amplified spontaneous emission (ASE) noise loading stage, constituted by an erbium-doped fiber amplifier (EDFA) and an optical filter (OF) with 1 nm bandwidth. Two TL pumps are coupled with the noisy data and injected into the SOA: the output converted data at λ_FWM_ are selected, and DPSK demodulation is made by using a standard delay-interferometer (DI). The dual co-polarized pumps allow FWM independence from input signal polarization and wavelength. In the system, which is suitable for photonic integration, wavelength conversion in a range up to 6 nm was measured [181].

SOAs, however, are limited in speed due to carrier dynamics, and other attempts were made of realizing an integrated all-optical regenerator by means of other devices. One of the earliest results was obtained by B.J. Eggleton’s group exploiting the strong Kerr nonlinearity of As_2_S_3_ chalcogenide glass waveguides (n_2_~2 × 10^−14^ cm^2^/W) [183]. The operation principle of the integrated 2R regenerator was based on a combination of nonlinear SPM-induced spectral broadening followed by spectral filtering; the device was constituted by a low loss As_2_S_3_ rib waveguide with integrated Bragg gratings. A nonlinear power transfer curve was demonstrated using 1.4 ps pulses, enough to suppress noise in an amplified transmission link [183]. Shortly thereafter, an SOI waveguide device was demonstrated to perform optical regeneration based on a Si nanowaveguide as the nonlinear medium for the SPM process integrated with a bandpass filter consisting of a waveguide ring resonator [184]. Compared with the chalcogenide device, which required ~50 W peak power, the SOI device operated at peak powers lower than 5 W. Since then, many other excellent results have been achieved; here, only a few of them are mentioned. On-chip four-level pulse–amplitude modulation (PAM-4) wavelength conversion and signal regeneration were demonstrated in a silicon waveguide, exploiting degenerate FWM [36]. Using a silicon nanowire waveguide, Geng et al. [185] experimentally demonstrated three-wavelength regeneration based on the clock-pump four-wave-mixing scheme. 

A limitation of silicon devices lies in nonlinear absorption (two-photon absorption, TPA): it can, however, be circumvented by using reverse-biased p-i-n diode structures implemented across the waveguide [186]. With this approach, phase regeneration for DPSK signals using a Si waveguide with a reverse-biased p-i-n junction as nonlinear medium and continuous-wave pumping was demonstrated, with high wavelength conversion efficiency [187]. The Si nano-rib waveguides had width, height, and slab height of 500 nm, 210 nm, and 50 nm, respectively; doped regions were created at a distance of 350 nm from the waveguide through implantation of boron and arsenic with concentrations of 10^18^ cm^−3^ for the p- and n- regions, respectively. Keeping in mind development of 5G and 6G networks, data transmission rates as high as 100 Gb/s at the user must be considered; correspondingly, intensity modulation with the NRZ-OOK format will likely be a good solution for optical access networks. Wen et al. [180] have, therefore, investigated the feasibility of all-optical regeneration of these signals using the FWM process in a silicon waveguide with reverse-biased p-i-n junction. The Si rib waveguide is 4 cm long, 450 nm wide, and 70 nm thick, respectively. The p- and n- regions of the junction were 3.6 µm wide and separated by 1.35 µm; they had been obtained by implantation of boron and phosphorus ions, respectively. The achieved conversion efficiency was as high as −12 dB, and all-optical regeneration of NRZ-OOK signals with rates of 50 Gb/s and 100 Gb/s were experimentally demonstrated [188]. For completeness, it has also to be mentioned that signal regeneration was obtained by exploiting the nonlinear properties of lithium niobate, e.g., using nonlinear wave mixing in cascaded periodically poled lithium niobate (PPLN) waveguides [189,190,191] and in lithium niobate on insulator (LNOI) structures [35].

Before concluding our discussion on all-optical signal regeneration, we summarize some challenges for achieving efficient all-optical regenerators: (1) as optical networks are adopting flexible and modulation format variable transceivers, any dependence on modulation format will reduce the efficiency and upgradability of the regenerators; therefore, optical regeneration should be modulation format and baud-rate-transparent. (2) Optical regeneration should improve the optical SNR by amplifying only the data signal and reducing the signal’s amplified spontaneous emission (ASE) noise arising from erbium-doped-fiber-amplifiers (EDFA). (3) The optical regenerator should mitigate any crosstalk and inter-symbol interference that arises in the different domains of the optical wave, such as wavelength, polarization, and spatial modes [50,51].

### 4.3. Nonlinear Sources

To generate SC from a chalcogenide glass fiber, a short pulse laser (i.e., fs laser) is often employed as the pump source operating at the NIR region where dispersion of chalcogenide glasses is positive. In SC generation under NIR pumping, the resultant spectra usually show limited bandwidth due to the limited pump power and materials dispersion. To this end, optical pumping at wavelengths longer than the zero-dispersion wavelength (ZDW) is often applied, where the dispersion of the chalcogenide glasses becomes negative. The SC generation is this case becomes much more efficient as the soliton effect becomes dominant, such as soliton splitting and self-frequency shift, which leads to much larger broadening of the SC spectra [192].

**Figure 15:**
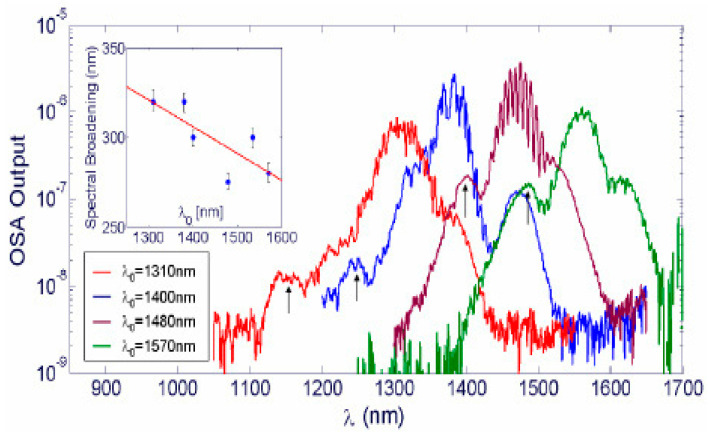
Supercontinuum generation in a 4.7 mm long silicon photonic wire waveguide for several input central wavelengths at P 0 ≈ 1W. The inset shows that spectral broadening increases as λ_0_ approaches the ZGVD wavelength of 1290 nm. Reprinted with permission from [193] © The Optical Society.

An experimental demonstration of a tunable continuum source in silicon photonic wires (SPWs) with a power-dependent broad spectrum was reported in [193]. As can be seen in Figure 15, a spectral broadening of more than 350 nm was observed upon propagation of ultrashort 1.3 μm wavelength optical pulses in a 4.7 mm long single-mode waveguide. Supercontinuum white light generation in plasmonic nanostructures, depending on the nonlocality of the electron response, has also been theoretically investigated [194].

Mode-locked lasers were initially used for comb generation. Thereafter, comb emission was demonstrated in continuous-wave (cw) laser-pumped resonators through cascaded third-order parametric processes [195]. In such Kerr resonators, a first pair of sidebands is generated around the pump frequency by cavity modulation instability or degenerate four-wave mixing (FWM); subsequently, cascaded four-wave mixing processes lead to formation, around the pump frequency, of a uniform frequency comb, where self- and cross-phase modulation act to compensate for the unequal cavity mode spacing induced by the group velocity dispersion (GVD) [196]. Because of the relatively low strength of third-order nonlinearity, generation of Kerr combs requires small interaction volumes and high-Q resonators. For these reasons, small resonators are particularly suited to reach broadband comb generation with quite moderate pump power [62]. Advancements in the fabrication technology of optical micro-cavities may enable realizing ultra-fast and stable optical clocks and pulsed sources with extremely high repetition-rates in the form of compact and integrated devices. In this framework, demonstration of planar high-Q resonators, compatible with silicon technology [197,198], has revealed a unique opportunity for these devices to provide entirely new capabilities for photonic-integrated technologies. 

**Figure 16:**
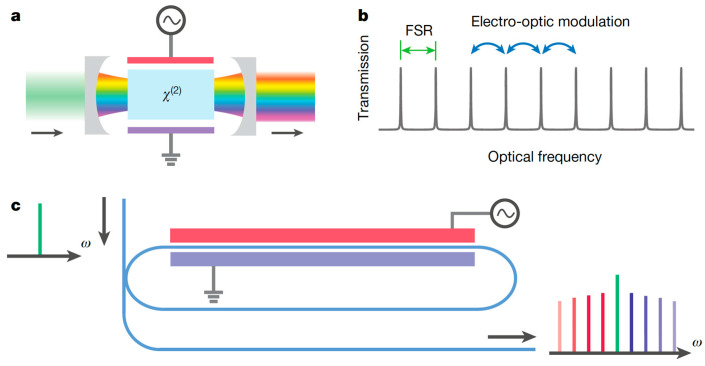
EO comb generator using a microring resonator. (**a**) Sketch of a bulk EO comb generator based on an EO (χ^(2)^) phase modulator inside a Fabry–Pérot resonator. A cw laser beam enters the resonator and an optical frequency comb is generated at the output. (**b**) EO comb generation principle; the microwave modulating signal has frequency equal to FSR of the Fabry–Pérot resonator. (**c**) Integrated EO comb generator, where a microring replaces the Fabry–Pérot resonator. The cw signal from input waveguide is coupled into the microring and EO-modulated at a frequency matching the FSR of the ring. The generated comb is then coupled back to the waveguide. Reproduced with permission from [199] © Springer Nature Ltd.

It can be recalled that electro-optic (EO) modulation, in materials with second-order nonlinearity, provides an interesting alternative to Kerr (third-order nonlinearity) resonators for generation of OFCs; the electrical controllability of EO combs guarantees greater versatility and also excellent comb stability and phase coherence. A microring resonator, with loaded Q~1.5 × 10^6^, may, therefore, be fabricated in a thin-film lithium niobate (TFLN) platform, which is also characterized by ultra-low-loss optical waveguides [199]. This solution is depicted in Figure 16; a microwave signal, with modulation frequency equal to the free spectral range (FSR) of the optical resonator, couples light between different resonator modes. The input cw laser light is, therefore, modulated, giving rise to sidebands at the modulation frequency, which are then recirculated to be modulated again. At the output, an EO comb can be measured that spans more frequencies than the entire telecommunications L-band (over 900 comb lines spaced about 10 gigahertz apart), with the prospect of enabling octave-spanning by proper dispersion engineering [199]. A disadvantage of this single-resonator device is the low (~0.3%) comb conversion efficiency due to the low coupling efficiency of the light from the input waveguide to the microring EO-driven resonance. This limit was overcome by the same research group by using two mutually coupled resonators, again realized in the TFLN platform: the fabricated device proven to be capable of generating an on-chip EO comb with a line spacing of 30.925 GHz, a pump-to-comb conversion efficiency of 30%, and a wide comb span of 132 nm [200].

Early attempts of micro-comb-based communications used simple on–off keying (OOK) modulation [201,202], where information is carried by the optical intensity. In this case, low comb intensity noise is essential for achieving high performance. Error-free transmission for each individual line of a low-noise micro-comb, transmitted over tens of kilometers of single-mode fiber, has been demonstrated with a power penalty of less than 0.5 dB [202].

Coherent data transmission, which typically poses stringent requirements on the spectral purity of the optical carrier, has also been demonstrated with phase-locked micro-combs. In the first work reported by Pfeifle et al. in 2014 [203], a data stream of 392 Gbit s^−1^ was encoded on six lines of a micro-comb using quadrature phase-shift-keying (QPSK) and 16-state quadrature amplitude modulation (16QAM). A second experiment demonstrated the feedback stabilization of the comb and transmission of a 1.44 Tbit s^−1^ data stream over up to 300 km with twenty comb lines. These results showed that micro-combs can indeed meet the highly demanding requirements of coherent communications and thus offer an attractive route towards chip-scale terabit/s transceivers [204].

## 5. Conclusions

Despite the difficulties, impressive progress has been made in on-chip nonlinear optical processing with favorable performance, paving the way to full integration of complete optical communication and processing systems on a monolithic or hybrid chip. As an example, an excellent result concerns the high device integration density achieved by using optimized racetrack microresonators based on ultra-low-loss multimode Si_3_N_4_ photonic circuits. The optimized design uses adiabatic Euler bends in place of circular bends; it enables a device footprint as small as 0.21 mm^2^, which can be very useful for several nonlinear photonic applications. Single-soliton generation of 19.8 GHz repetition rate was demonstrated; the soliton spectrum showed a 3-dB bandwidth of 16.3 nm, corresponding to a pulse duration of 156 fs [3]. Even better prospects exist for silicon carbide, which now appears able to fully compete with more mature nonlinear materials; as a proof, design and demonstration of octave-spanning microcombs have been reported in a 4H-SiC-on-insulator microring resonator with 36 µm radius [136].

All-optical computing, on the other hand, still presents many challenges due to the difficulty of implementing efficient all-optical logic devices. Photonic crystals seem to offer a suitable approach to realization of these devices, but, up to now, groundbreaking results have not been achieved. Other approaches make use of nonlinear optical fibers and semiconductor optical amplifiers, but, in both cases, size is an obstacle to chip integration. Plasmonic logic gates would have a desirable nanometer size, but their large optical loss makes them also unsuitable for use in integrated chips. As has been shown by various examples, PICs fabricated on a silicon-on-insulator platform may exhibit low power consumption and high transmission efficiency but occupy a relatively large footprint. A solution has, therefore, still to be searched. However, based on the linear interference approach, it is worth reporting here a very recent result obtained using a design based on topology optimization [205] and leading to implementation of an SOI chip containing seven major logic gates (AND, OR, NOT, NAND, NOR, XOR, and XNOR) and a half adder, with a footprint of only 1.3 × 4.5 μm^2^ [205].

The evolving nano-devices would enable emerging advanced applications not only in optical processing and computing but also in metrology, single-molecule sensing, imaging, microscopy, mid-infrared photonics, terahertz generation, microwave photonics, and bio-medicine. We expect that progress in nonlinear integrated photonics will occur along three paths: one involving new materials and new physics in nonlinear interactions; a second devoted to improving device performance using well-known and innovative guided-wave nonlinear optical schemes; and a third addressing new perspectives in quantum photonics. In this regard, being impossible to adequately cover the achievements in quantum optics in this short review, we just mention a few examples, all exploiting the exceptional characteristics of microring resonators, either on a silicon platform [206,207] or a 4H-SiC-on-insulator platform [208]. Quantum technologies may well exploit other materials as well, and we refer interested readers to an extended review on the 2023 roadmap for materials for quantum technologies, published in January 2023 [209].

Returning to use of Si or SiC, Guo et al. experimentally demonstrated nonclassical optical bistability in a photon-pair source using cw-laser-pumped spontaneous FWM in an all-pass microring resonator with 100 µm radius fabricated on an SOI platform [206]. Using similar microring (110 µm radius) with Q~5 × 10^4^ and free spectral range (FSR)~100 GHz, matching the standard International Telecommunication Union (ITU) frequency grid to enable multichannel photon-pair generation compatible with commercial WDM devices, the same research group showed how frequency correlation can be applied to high-dimensional encoding for parallel quantum key distribution (QKD) [207]. 

More recently, various works have underlined the strong potential of 4H-SiC platform for on-chip quantum photonics; it was also demonstrated that implantation of silicon vacancy centers in SiC does not deteriorate their intrinsic spin-optical properties [210]. This result makes feasible development of large-scale quantum networks based on integrated quantum computational clusters with efficient spin–photon interfaces. Lukin et al. reported both strong enhancement of emission from a single color center in a nanophotonic cavity and efficient nonlinear frequency conversion in the same platform, also demonstrating full compatibility of thin-film SiC with industry-standard nanotechnologies and foundry production [208]. Still using a SiC microring structure, Guidry et al. studied the underlying quantum processes of soliton microcombs, investigating the quantum formation dynamics of dissipative Kerr soliton (DKS) states and their quantum correlations [211].

Obviously, theoretical advances are being made as well; as an example, Nozaki et al. have now (February 2023) published a paper where they propose to use a resonator with only passive optical components to scale up stimulated emission of polarization-entangled photon pairs [212]; they present only a proof-of-principle experimental demonstration with a double-pass polarization Sagnac interferometer and periodically poled KTP crystal, but use of waveguide structures should lead to realization of highly efficient and bright quantum-entangled photon sources needed for quantum information technologies.

## Figures and Tables

**Table 1:** Comparison of continuous-wave FWM nonlinear efficiency conversion ηNL in some integrated devices.

Geometry	Material	On Chip Power (mW)	Footprint (μm^2^)	Q_avg_	η_NL_(dB)
PhC on SOI [165]	InGaP	3	39	55,000	–12
Ring [166]	AlGaAs-O-I	7	929	44,000	–12
Ring-CROW(*) [167]	Si	100	4140	x	–21
Ring [168]	Graphene oxide on Hydex glass	158	x	50,000	–35
Ring [169]	Hydex	6	5730	10^6^	–36
Ring [170]	Grahene	8	314	9000	–37
